# Utilizing glycine N-methyltransferasegene knockout mice as a model for identification of missing proteins in hepatocellular carcinoma

**DOI:** 10.18632/oncotarget.23064

**Published:** 2017-12-07

**Authors:** Ming-Hui Yang, Wan-Jou Chen, Yaw-Syan Fu, Bin Huang, Wan-Chi Tsai, Yi-Ming Arthur Chen, Po-Chiao Lin, Cheng-Hui Yuan, Yu-Chang Tyan

**Affiliations:** ^1^ Center for Infectious Disease and Cancer Research, Kaohsiung Medical University, Kaohsiung, Taiwan; ^2^ Department of Medical Imaging and Radiological Sciences, Kaohsiung Medical University, Kaohsiung, Taiwan; ^3^ Department of Biomedical Science and Environmental Biology, Kaohsiung Medical University, Kaohsiung, Taiwan; ^4^ Department of Medical Laboratory Science and Biotechnology, Kaohsiung Medical University, Kaohsiung, Taiwan; ^5^ Graduate Institute of Medicine, College of Medicine, Kaohsiung Medical University, Kaohsiung, Taiwan; ^6^ Department of Chemistry, National Sun Yat-sen University, Kaohsiung, Taiwan; ^7^ Mass Spectrometry Laboratory, Department of Chemistry, National University of Singapore, Singapore; ^8^ Institute of Medical Science and Technology, National Sun Yat-sen University, Kaohsiung, Taiwan; ^9^ Department of Medical Research, Kaohsiung Medical University Hospital, Kaohsiung, Taiwan

**Keywords:** glycine N-methyltransferase, hepatocellular carcinoma, missing protein, proteomics, human proteome atlas

## Abstract

Glycine *N*-methyltransferase is a tumor suppressor gene for hepatocellular carcinoma, which can activate DNA methylation by inducing the *S*-adenosylmethionine to *S*-adenosylhomocystine. Previous studies have indicated that the expression of Glycine *N*-methyltransferase is inhibited in hepatocellular carcinoma. To confirm and identify missing proteins, the pathologic analysis of the tumor-bearing mice will provide critical histologic information. Such a mouse model is applied as a screening tool for hepatocellular carcinoma as well as a strategy for missing protein discovery. In this study we designed an analysis platform using the human proteome atlas to compare the possible missing proteins to human whole chromosomes. This will integrate the information from animal studies to establish an optimal technique in the missing protein biomarker discovery.

## INTRODUCTION

The completion of the human genome project that decoded more than 20,000 protein-coding genes has inspired enthusiastic efforts toward complete mapping of the human proteome to understand human biology. The missing proteins were originally proposed in 1994 by scientists from Singapore. Recently, A*STAR’s Institute of Molecular and Cell Biology (IMCB) have shown new evidence that p21 activated protein kinases (PAK) affects a cancer associated protein that scientists have been studying for years. Their latest findings were published recently in Molecular Cell and have shown 4228 missing proteins in human chromosomes. After that, subsequent studies have addressed various diseases [1–3].

The Human Proteome Project (HPP) is an international project organized by the Human Proteome Organization (HUPO), which is designed to map the entire human proteome in a systematic effort, using three major techniques: mass spectrometry (MS), antibodies (Abs), and the knowledgebase (KB) [4, 5]. It consists of two major programs: the chromosome-based HPP (C-HPP) and the biology/disease HPP (BD-HPP) by which can expand our understanding of the human proteome of each gene on each chromosome and important biology/disease-focused research. The Chromosome-Centric Human Proteome Project (C-HPP) aims to catalogue proteins as gene products encoded by the human genome in a chromosome-centric manner and to characterize their isoforms and functions. [6].

In the past, researchers have used the state-of-the-art high throughput proteomic technology, but major difficulties have arisen in the detection of a set of proteins, termed “missing proteins” [7]. Missing proteins were defined as proteins predicted to be encoded from the gene or transcriptomic analysis but with no available protein expression evidence or high degrees of confidence from mass spectral detection, antibody-capture, 3D structures (X-ray or NMR), or Edman sequencing [8]. The current list of coding genes for missing proteins includes genes having transcript expression evidence, genes inferred from homologous proteins in related species, genes hypothesized from gene models, and “dubious” or “uncertain” genes [1]. Thus, missing proteins have not been experimentally validated, but have been detailed extensively including low abundance, time- or stress-dependent or organ-specific expression, particular physicochemical properties (hydrophobicity, amino-acid composition), or protein instability [9]. Several of these issues can arise simultaneously or sequentially, resulting in complicated situations. Previous studies have indicated that the production of systematically missed proteins might be restricted to unusual organs or cell types [10].

These proteins lacking experimental evidence were obtained by mass spectrometry or antibody-based detection, and their existence is based on bioinformatic predictions or transcriptomic analyses. There are several reasons for the difficulties in the detection of these proteins, including incorrect gene annotation, very low abundance, absence of expression in a given tissue, expression only in rare samples, and unfavorable structure (or cleavage sites) for MS studies such as instability or heterogeneity [11, 12]. Furthermore, the neXtProt is utilized as the reference database for the annotation of human proteins in the beginning period of the C-HPP [13, 14]. This database assigns experimental evidence to each human protein using a scale with five levels, from PE1 (experimental evidence at protein level), PE2 (experimental evidence at the transcript level), PE3 (protein inferred from homology), PE4 (protein predicted) to PE5 (uncertain protein). The missing proteins are annotated as PE2 to PE4 [15, 16].

Glycine N-methyltransferase (GNMT) is a protein with multiple functions that activates genetic stability by (a) regulating the ratio of *S*-adenosylmethionine (SAMe) to *S*-adenosylhomocystine (SAH) and (b) acting as a major folate-binding protein. The human GNMT gene was cloned and characterized as being ocated on the short (p) arm of chromosome 6 at position 12. More precisely, the *GNMT* gene is located from base pair 42,939,414 to base pair 42,963,879 on chromosome 6. For mouse, the *GNMT* gene was located on chromosome 17.

The GNMT gene was also considered as a tumor suppressor gene. Deletion of *GNMT* in mice leads to the development of fatty liver, fibrosis and hepatocellular carcinoma as a result of sustained increased levels of SAMe in livers. Liver regeneration is also impaired in GNMT knockout (GNMT^−/−^) mice due to increased SAMe levels. In addition, increased SAMe levels lead to an increase in methylation of DNA and histones [17].

A recent study identified a novel tumorigenic mechanism of dysregulation of phosphatidylinositol 3,4,5-trisphosphate-dependent Rac exchanger 2 protein (PREX2) expression in a tumor environment where GNMT expression is inhibited. As shown in clinical hepatocellular carcinoma (HCC) specimens, the expression of GNMT in a tumor was much lower than that in the tumor adjacent area [18]. The GNMT may enhance ubiquitination of PREX2 and a consequent increased PREX2 degradation through association with HectH9, and regulation of cell proliferation in normal liver. In GNMT-inhibited liver environment, lower association between E3 ubiquitin-protein ligase HUWE1 (HectH9) and PREX2 leads to reduced ubiquitination of PREX2 and a subsequent dysregulation of PREX2 expression. PREX2 overexpression results in increased serine/threonine-protein kinases (AKT) signaling, dysregulated cell proliferation and HCC development. GNMT, PREX2 and its variant mutants may serve as new therapeutic targets of HCC.

A driving force in proteomics is the discovery of biomarkers. Missing proteins that change in concentration or state in association with a specific biological process or disease are candidates. Determination of concentration changes, relative or absolute, is fundamental to the discovery of valid biomarkers [19]. Missing proteins may coordinate various cellular processes in tumor cells, including growth, division, differentiation, apoptosis, migration, metastasis, angiogenesis and adhesion; and thereby contribute to the growth and spread of the tumor cells [20]. Thus, the identification of missing proteins in tumor cells provides potential biomarkers closely related to carcinogenesis. Moreover, disease-specific protein biomarkers allow us to define the prognosis of the disease and gain deep insight into disease mechanisms by which proteins play a major role. The database we generated provides information both on the identities of proteins present in tumor cells and a potential diagnostic biomarker for cancer.

In this study, we used the mass spectrometry technique as an analytical method for determining cancer biomarkers on tumor missing proteins. The GNMT^−/−^ mouse model was used to identify the proteins related to tumor progression. A database is created for the diversity and relative abundance of various missing proteins found in the animal model. The database provides not only information on the nature of missing proteins present in the tumor, but also potential protein diagnostic markers to be examined in further investigations.

## RESULTS AND DISCUSSION

GNMT, also known as a 4S polycyclic aromatic hydrocarbon binding protein, acted as a major folate binding protein and increased the ratio of SAM to SAH by catalyzing sarcosine from glycine. In the previous reports, it has been indicated that GNMT expression was inhibited in HCC and was a tumor susceptibility gene for liver cancer [21]. In this study, the GNMT^−/−^ mouse model was used to study the missing proteins in the GNMT^−/−^ mouse and to search the early diagnosis biomarkers for hepatocarcinogenesis. In order to assess the liver function and the formation of hepatocellular carcinoma/adenoma, the detection of aminotransferase levels and pathological diagnosis were used in this experiment.

### Serum ALT and AST levels

**Figure 1:**
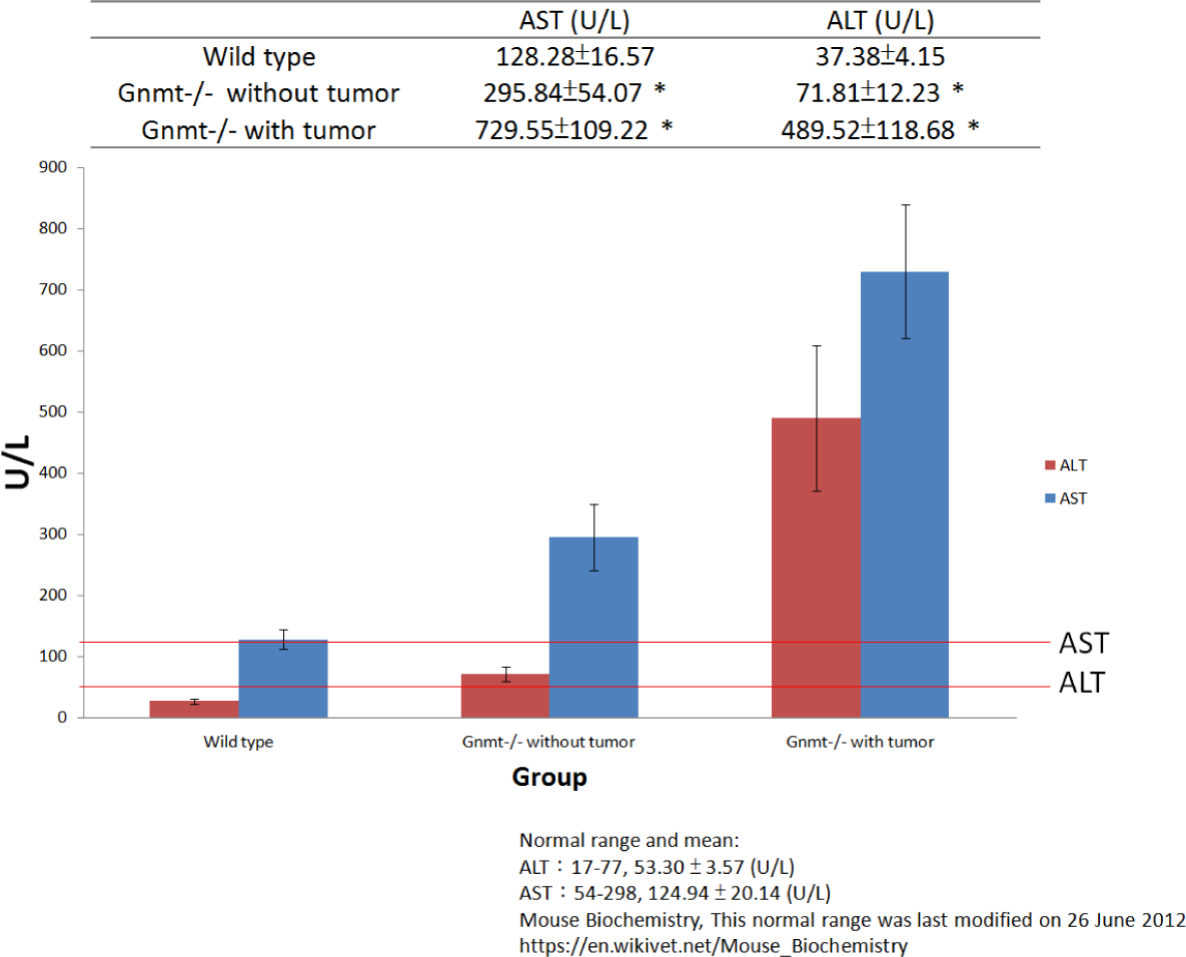
Serum alanine aminotransferase (ALT) and aspartate aminotransferase (AST) level measurements showed that the mean AST and ALT levels in the GNMT^−/−^ with tumor mice were significantly higher than that in the wild-type mice (*n* = 10, mean ± SD, *P* < 0.05)

Many different proteins and enzymes are produced from the liver; however, two major liver enzymes, ALT and AST, can provide information of liver function. AST is an enzyme found in liver, muscle and heart, and ALT is found in the liver and kidneys. When liver cells are damaged, they release AST and ALT into blood. The ratio of AST to ALT level can help determine for liver damage. In the group of GNMT^−/−^ mice without tumor, the AST to ALT ratio was greater than 2:1, which was suggestive of fibrosis or chronic hepatitis. High levels of AST and ALT are often found in people with liver damage, which may be a sign of liver tumor formation. The results from serum AST and ALT level measurements showed that the mean AST and ALT levels in the GNMT^−/−^ with tumor mice were significantly higher than that in the wild-type mice (Figure 1, *P* < 0.05). The pathological findings also indicated hepatocellular carcinoma/adenoma.

### Pathological findings for Gnmt^−/−^ mice

**Figure 2:**
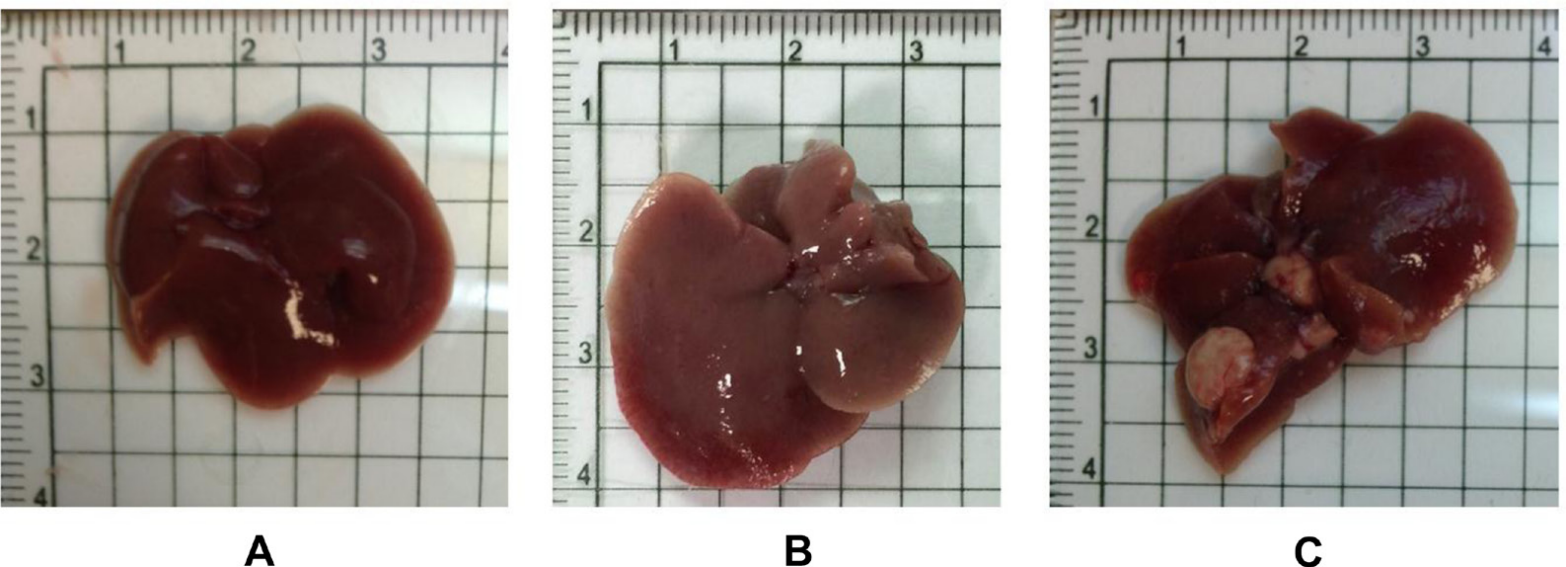
The overall appearances of the liver organs from both wild-type and Gnmt^−/−^ mice were relatively normal at the age of 12 months (**A**: Wild Type, **B**: Gnmt^−/−^). Multiple white tumor nodules were found in the livers of Gnmt^−/−^ mice of 16 months (**C**).

The mouse liver appearances from groups of wild-type and GNMT^−/−^ without tumor were relatively normal at the age of 12 months (Figure 2A and 2B). Multiple white tumor nodules approximately 5 mm in diameter were found in the livers of GNMT^−/−^ mice of 16 months (Figure 2C).

Histologically, altered hepatocellular foci (AHF) were presumptive preneoplastic lesions that can vary from barely perceptible to cytomorphologically and tinctorially discrete lesions. Morphological features and classification of foci in mice were generally classified as basophilic, eosinophilic, clear, or mixed cell foci based on their cytological features in H&E-stained sections. In these livers from GNMT^−/−^ mice, predominant types were clearly AHF.

**Figure 3:**
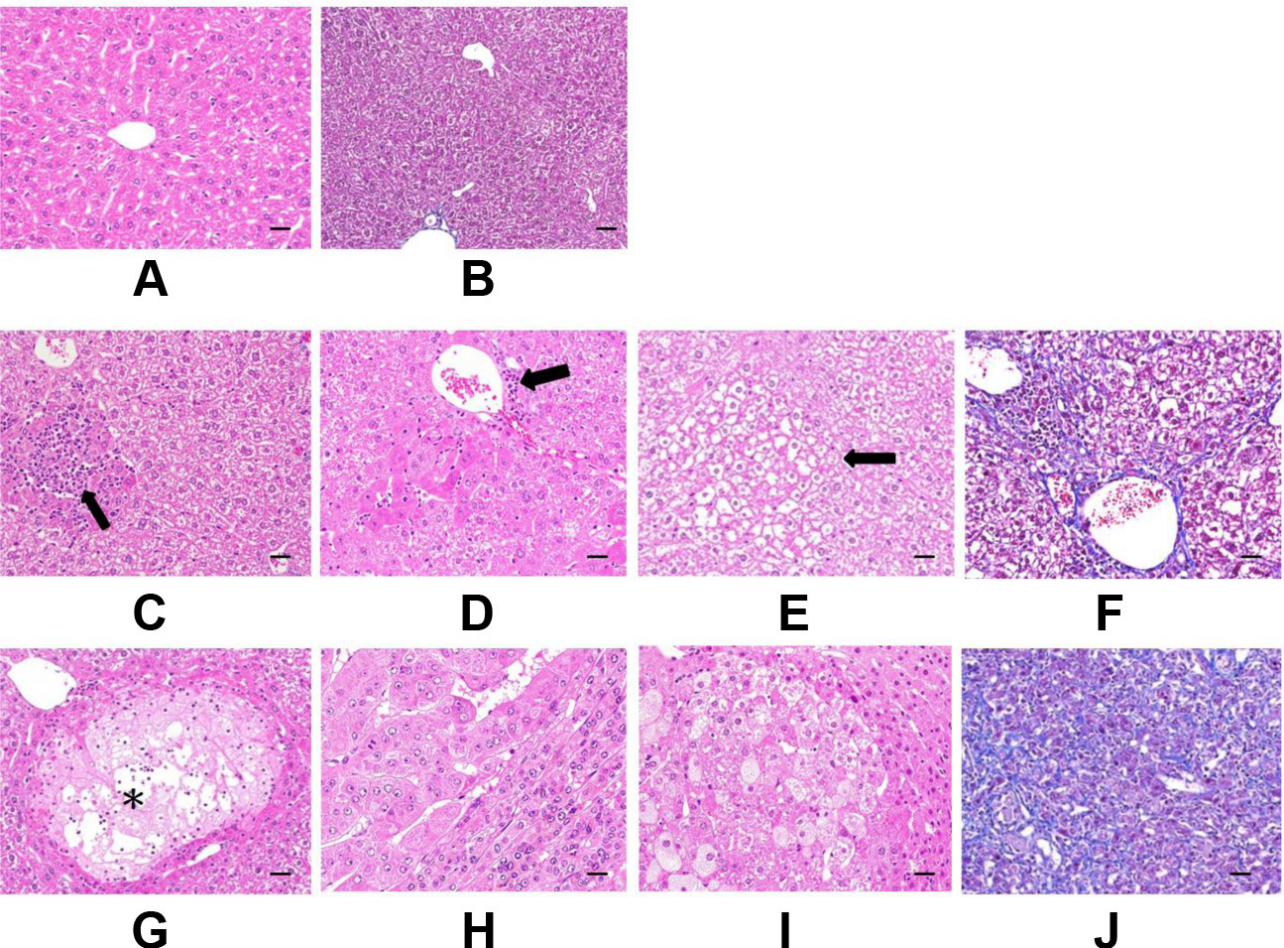
Histopathological examination of mice liver (**A** and **B)** are Reference photographs. (A) 4 months old C57BL/6 male, hematoxylin and eosin (H&E) stain, no glycogen accumulation. (B) 9 months old C57BL/6 male, Masson’s Trichrome stain, no fibrosis. C to F are GNMT^−/−^ without tumor. (**C**) Focal hepatocellular necrosis with inflammatory cell infiltration (arrow) and glycogen accumulation. (**D)** Glycogen accumulation (grade 2), hepatocellular hypertrophy, inflammatory cell infiltration (arrow). (**E**) Clear cell focus of cellular alteration (arrow). Glycogen accumulation. (**F**) Fibrosis expansion without septa formation. G to J are GNMT^−/−^ with tumor. (**G**) Focal hepatocellular hydropic degeneration and necrosis with inflammatory cell infiltration (^*^). Glycogen accumulation. (**H**) Hepatocellular carcinoma with adjacent normal hepatocytes compression. (**I**) Hepatocellular adenoma with adjacent normal hepatocytes compression. Cytoplasmic vacuolation is prominent in tumor cells. (**J**) Diffuse fibrosis of hepatic subcapsule. C to E and G to I were stained by H&E stain. F and J were stained by Masson’s Trichrome stain. The scale bars indicate 30 μm, 400×.

**Figure 4:**
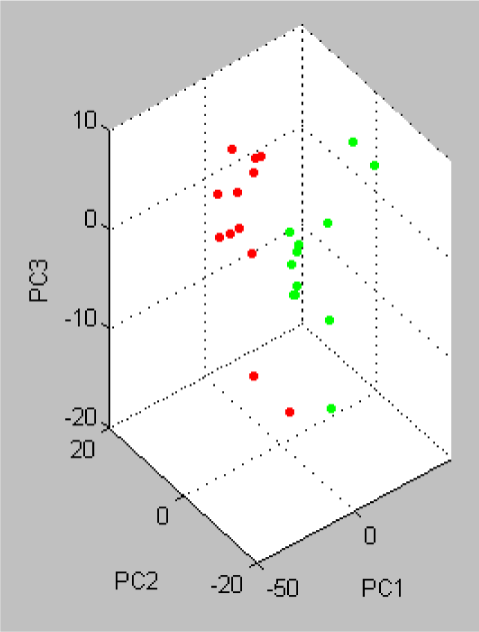
Principle Component Analysis (PCA) of the analyzed the wild type and GNMT^−/−^ mice using the software tool SPSS Each dot stands for a displayed data indicating the calculated cluster membership in which each dot represents one of the wild type mice (red) or GNMT^−/−^ mice (green). A clear separation of the wild type mice and GNMT^−/−^ mice is apparent.

**Figure 5:**
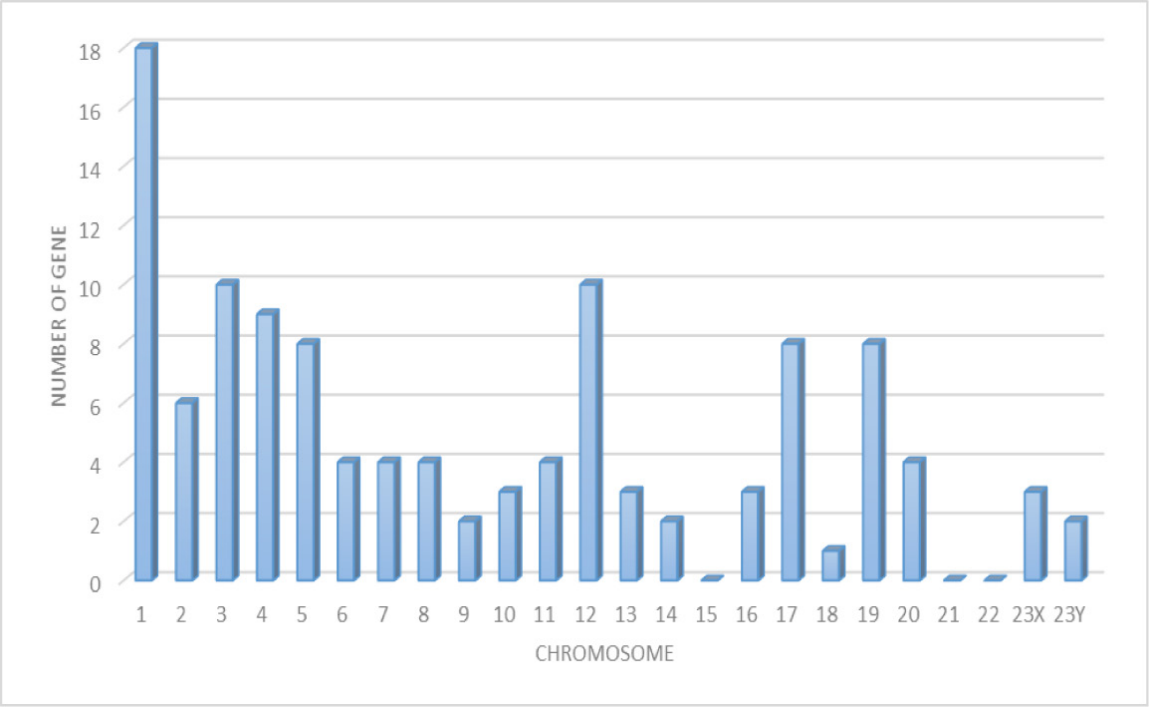
The distribution of missing proteins according to the chromosomal location of their genes (retrieved from the Human Protein Atlas databases (HPA)) The human chromosome assignation and protein evidence status of missing proteins correspond and identified in the liver of mouse.

**Figure 6:**
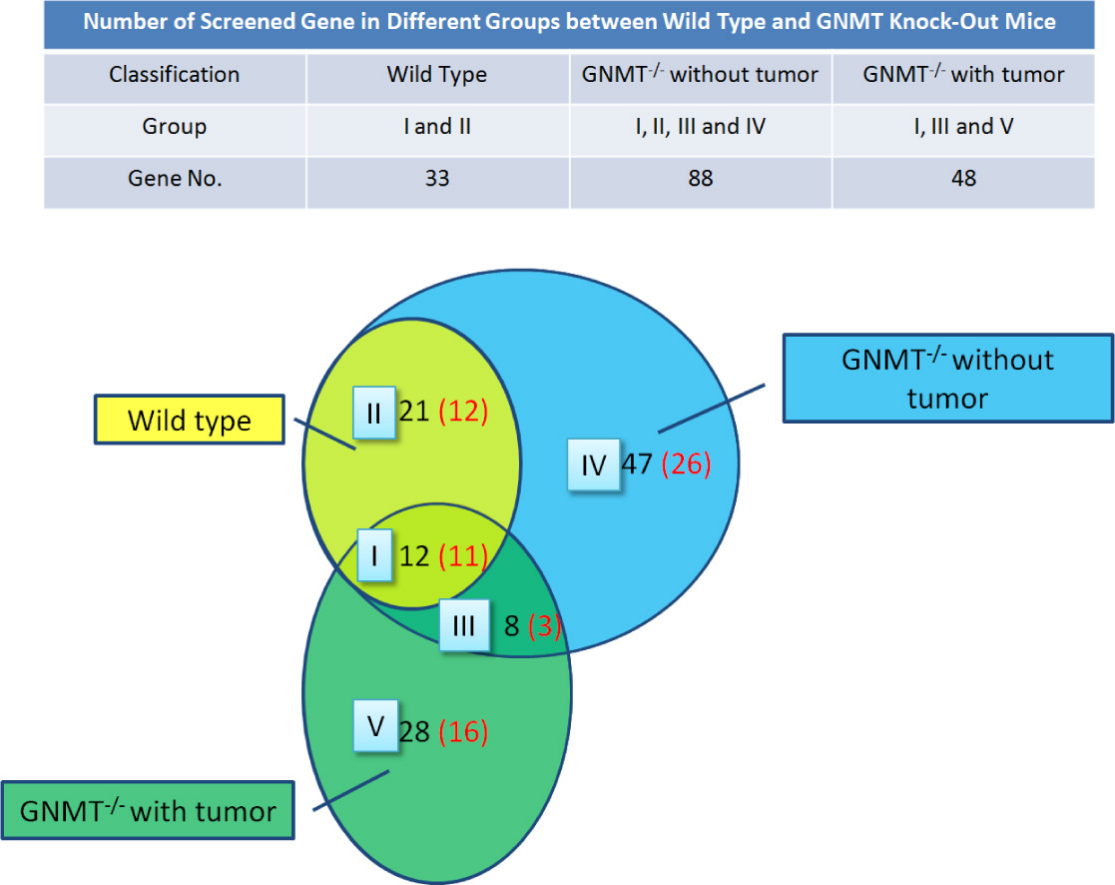
Missing proteins detected in the mouse liver proteome Screen genes (in black) and missing protein (in red) were overlapped between the three mice groups. The evidences of protein level were screened in UniProt and the Human Protein Atlas (HPA) databases.

Histopathological examination results of the liver tissues are presented in Figure 3. Figure 3A and 3B are reference photographs. No glycogen accumulation or fibrosis was found. Figure 3C to 3F are photographs from the group of GNMT^−/−^ without tumor; Figure 3G–3J are photographs from GNMT^−/−^ with tumor group. Some microscopic lesions were observed in liver tissues consisting of: (1) hepatocellular hypertrophy, inflammatory cell infiltration in the liver and glycogen accumulation (Figure 3C and 3D); (2) focus of hepatocellular alteration (clear cell foci, mixed cell foci, and eosinophilic foci) (Figure 3E); (3) hepatocellular degeneration/necrosis with inflammatory cell infiltration (Figure 3G); (4) hepatocellular carcinoma (Figure 3H); (5) hepatocellular adenoma (Figure 3I); (6) hepatic fibrosis expansion of the liver (Figure 3F and 3J). A nodule of the abdominal wall was observed in the (Figure 3E) mouse. Histopathologically, the nodule appeared to be severe pyogranulomatous peritonitis. There are different pathological symptoms in the GNMT^−/−^ with or without tumor groups. Because some histological lesions have been generated before the tumor formation (Figure 3C–3F), it was not able to evaluate the relationship with hepatocellular carcinoma or hepatocellular adenoma. In another word, the pathological symptoms before tumor formation do not determine the type of tumor formation. In Figure 3G–3J, tumor formation and tissue necrosis are evident. In addition, using H&E staining to distinguish hepatocellular adenoma from hepatocellular carcinoma in mouse samples is complicated.

Hepatocellular adenomas are usually larger than foci with distinct borders, and cause compression of surrounding parenchyma. They are composed of mildly to moderately pleomorphic hepatocytes that are of normal sizes or slightly larger than normal ones. Hepatocellular carcinomas poorly demarcate from adjacent tissues, and entire liver lobes can be involved. They show several morphological alterations including increased cell volume and chromatin disorganization, and also may be round and swollen to about twice the size of normal cells. They also lose hepatic cell borders, hepatic plate arrangement and show pale staining of cytoplasm.

Cell swelling was diffuse and presented with granular or lacy appearing cytoplasm. GNMT^−/−^ mice were observed to have clear cell foci; the foci may be potential precursors of neoplasm formation. Vacuoles were often seen to lack uniformity of size or discrete outlines. The swollen hepatocytes with extensive cytoplasmic vacuolation were from glycogen accumulation. Glycogen deposition may result in a vacuolated cytoplasmic appearance. Based on the pathological findings presented in this study, we can find some microscopic lesions in tissues of the wild type mice and GNMT^−/−^ mice. Several GNMT^−/−^ mice appeared to have increased collagen in the liver (grade 1-2 hepatic fibrosis).

PCA, an additional statistical tool, was included in Statistical Product and Service Solutions (SPSS) and applied to the analyzed datasets to visualize the homogeneity and heterogeneity of the protein concentrations. PCA reduces the variables of a complex dataset on the basis of different statistical tests. In this study, wild type mice and GNMT^−/−^ mice were displayed in three-dimensional PCA in Figure 4. Each dot stands for a displayed data indicating the calculated cluster membership in which each dot represents one of the wild type mice (red) or GNMT^−/−^ mice (green). A clear separation of the wild type mice and GNMT^−/−^ mice is apparent.

In this study, GNMT^−/−^ mice were chosen as the animal model for deep profiling of HCC missing proteins. We divided the mice into three groups: wild type, GNMT^−/−^ without tumor and GNMT^−/−^ with tumor. Hepatic proteins from those groups were extracted, digested and applied to a nano-UPLC-ESI-MS/MS system for fragmentation patterns of tryptic peptides. Each sample was subjected to 3 replicate runs, and duplicate peptide ratios among these three groups were ranging from 73% to 84%, with an average of 80.2%.

There were thousands of unique peptides identified in pooled samples. These proteins were identified at minimal confidence levels as only one unique peptide sequence was matched. The peptides identified from MS/MS spectra were carried out using our search program made by JAVA programming with the gene expression sequences downloaded from the HPA web. A missing protein was positively identified when two or more product ion mass spectra of peptides matched to the sequences with 100% of a missing protein in the database. By using our search program with gene expression sequences from the HPA web, three groups of 33, 88 and 48 proteins each, were uniquely identified with higher confidence levels (at least two unique peptide sequences matched.)

In this study, we compared the peptide expression of mouse livers from wild type, GNMT−/− without tumor and GNMT−/− with tumor by missing protein database. Many of these missing proteins were hard to be identified due to low abundance, poor solubility, or indistinguishable peptide sequences within protein families. Under a more stringent condition, positive protein identifications were considered when two or more unique peptides were identified. A total of 116 proteins were present in the three groups. Figure 5 shows the number of proteins with known chromosome locations. However, as many as 68 of the missing proteins, consisting of about 58.6%, were detected from data in the liver samples (listed in Supplementary Table 1). Among 68 missing proteins identified, 22 of them (32.2%) are known to be membrane protein, 18 are known to be nucleus protein. A few cytoplasm, secreted and intracellular proteins were also identified. A considerable portion of the identified proteins (16.1%) has not been reported for their cellular locations. According to the Human Genome Program Report [22], it has been demonstrated that there are similar genetic and homology aspects of the superficially dissimilar human and mouse species. The similarity was such that human chromosomes can be cut, and then reassembled into a reasonable approximation of the mouse genome. Thus, we want to use the mouse tumor model to project the corresponding chromosomes to humans and to solve the correlation between missing proteins with human chromosomes.

We used the UniProt databases (http://www.uniprot.org/uniprot/) proteomics server of the Swiss-Prot/TrEMBL to explore what known molecular functions and biological processes of the identified proteins had been reported in the literature. Supplementary Table 1 shows the biological process and molecular functions with certain reported known of the 68 proteins. Among 68 proteins, most of them (20 proteins, 29.4%) were related to DNA binding and transcription process. Binding of transcription factors to DNA is the supporting machinery for transcriptional activation or inhibition in tumor. Those proteins may play a role in the development and progression of the cancer phenotype. Some proteins still had no prior functional information reported.

The object is to study the differences of protein expression among wild type mice, GNMT gene knock-out mice with or without hepatic tumor. In the Figure 6, the proteins identified in the group IV were suspected protein before tumor formation, while groups III and V were likely to be associated with tumors. Proteins from GNMT−/− without tumor mice may be related to the induction of cancer and dismissed after tumor incidence. Proteins from GNMT−/− with tumor mice may be tumor-specific proteins, which should not be detected before tumor incidence. According to the results of this study, a more appropriate explanation is the expressions of missing proteins within group IV were decreasing after tumor formation in GNMT−/− mouse livers. On the other hand, the proteins within group V were increasing in GNMT−/− mouse livers during and after tumor formation. From among 83 proteins in the group III to V, 41 proteins were reported and related to tumorigenesis, in which 22 proteins were confirmed as missing proteins with using only the evidence at transcript level or protein prediction (Table 1). As the result in Figure 6, those missing proteins in group III to V may be some key proteins about the tumorigenesis.

**Table 1:** Summary of 41 proteins corresponding to tumorigenesis in group III to V

Group	UniProtKB Entry	Chromosome	Gene Name	UniProt protein name	Gene ID	Protein evidence
III	P0CG40	2	SP9	Transcription factor Sp9	ENSG00000217236	Evidence at protein level
	Q6ZWH5	3	NEK10-016	Serine/threonine-protein kinase Nek10	ENSG00000163491	Evidence at protein level
	Q7Z407	8	CSMD3-006	CUB and sushi domain-containing protein 3	ENSG00000164796	Evidence at protein level
IV	Q8N9H9	1	C1ORF127-001	Uncharacterized protein C1orf127	ENSG00000175262	Evidence at transcript level
	Q8TAY7	1	FAM110D	Protein FAM110D	ENSG00000197245	Evidence at transcript level
	Q6UY18	1	LINGO4	Leucine-rich repeat and immunoglobulin-like domain-containing nogo receptor-interacting protein 4	ENSG00000213171	Evidence at transcript level
	Q8N7P1	1	PLD5-006	Inactive phospholipase D5	ENSG00000180287	Evidence at transcript level
	A6NCL1	3	GMNC-001	Geminin coiled-coil domain-containing protein 1	ENSG00000205835	Evidence at protein level
	Q6ZWH5	3	NEK10-015	Serine/threonine-protein kinase Nek10	ENSG00000163491	Evidence at protein level
	Q6ZU67	4	BEND4-001	BEN domain-containing protein 4	ENSG00000188848	Evidence at protein level
	Q4ZJI4	4	SLC9B1-002	Sodium/hydrogen exchanger 9B1	ENSG00000164037	Evidence at protein level
	Q9H1J5	5	WNT8A	Protein Wnt-8a	ENSG00000061492	Evidence at transcript level
	Q96LW1	5	ZNF354B	Zinc finger protein 354B	ENSG00000178338	Evidence at transcript level
	Q8IXS0	6	FAM217A-001	Protein FAM217A	ENSG00000145975	Evidence at transcript level
	C9J798	7	RASA4B-201	Ras GTPase-activating protein 4B	ENSG00000170667	Protein inferred from homology
	Q8TE58	11	ADAMTS15	A disintegrin and metalloproteinase with thrombospondin motifs 15	ENSG00000166106	Evidence at transcript level
	Q8N4L1	11	TMEM151A	Transmembrane protein 151A	ENSG00000179292	Evidence at protein level
	Q9H2C1	12	LHX5	LIM/homeobox protein Lhx5	ENSG00000089116	Evidence at transcript level
	Q96LU7	12	MYRFL-003	Myelin regulatory factor-like protein	ENSG00000166268	Evidence at transcript level
	P46721	12	SLCO1A2	Solute carrier organic anion transporter family member 1A2	ENSG00000084453	Evidence at protein level
	Q7RTS6	17	OTOP2-201	Otopetrin-2	ENSG00000183034	Evidence at transcript level
	Q6ZSJ9	17	SHISA6-003	Protein shisa-6 homolog	ENSG00000188803	Evidence at protein level
	Q8NFU1	19	BEST2-002	Bestrophin-2	ENSG00000039987	Evidence at transcript level
	Q9BTN0	19	LRFN3-001	Leucine-rich repeat and fibronectin type-III domain-containing protein 3	ENSG00000126243	Evidence at protein level
	Q9HCL3	19	ZFP14-001	Zinc finger protein 14 homolog	ENSG00000142065	Evidence at transcript level
	P25100	20	ADRA1D-001	Alpha-1D adrenergic receptor	ENSG00000171873	Evidence at protein level
	Q8TDG2	23×	ACTRT1	Actin-related protein T1	ENSG00000123165	Evidence at protein level
	Q5QGS0	23×	KIAA2022-001	Protein KIAA2022	ENSG00000050030	Evidence at transcript level
	Q96NR3	23×	PTCHD1-201	Patched domain-containing protein 1	ENSG00000165186	Evidence at transcript level
V	A6NM62	1	LRRC53	Leucine-rich repeat-containing protein 53	ENSG00000162621	Protein predicted
	O75325	1	LRRN2-003	Leucine-rich repeat neuronal protein 2	ENSG00000170382	Evidence at transcript level
	A6NKT7	2	RGPD3-201	RanBP2-like and GRIP domain-containing protein 3	ENSG00000153165	Protein predicted
	Q9Y691	3	KCNMB2-001	Calcium-activated potassium channel subunit beta-2	ENSG00000275163	Evidence at protein level
	Q86VZ2	3	WDR5B-001	WD repeat-containing protein 5B	ENSG00000196981	Evidence at protein level
	Q15270	4	NKX1-1-001	NK1 transcription factor-related protein 1	ENSG00000235608	Evidence at transcript level
	Q16594	5	AK6	Transcription initiation factor TFIID subunit 9	ENSG00000273841	Evidence at protein level
	A6NJ46	8	NKX6-3-001	Homeobox protein Nkx-6.3	ENSG00000165066	Evidence at transcript level
	Q96LD1	8	SGCZ-001	Zeta-sarcoglycan	ENSG00000185053	Evidence at transcript level
	O76050	10	NEURL1-001	E3 ubiquitin-protein ligase NEURL1	ENSG00000107954	Evidence at protein level
	Q01851	13	POU4F1	POU domain, class 4, transcription factor 1	ENSG00000152192	Evidence at protein level
	Q9UBN1	17	CACNG4-001	Voltage-dependent calcium channel gamma-4 subunit	ENSG00000075461	Evidence at protein level

Twenty-two proteins were confirmed as missing proteins with evidence at transcript level or protein predicted.

In this study we attempted to find the possible missing protein in the GNMT−/− cancer mouse model, and those cancer-related proteins which were distributed on different chromosomes. It is an interesting phenomenon that there are three on the X chromosome and none on the Y chromosome. According to a previous report, the hypermutation of inactive X chromosomes is an early and frequent feature of tumorigenesis caused by DNA replication stress in abnormal proliferating cells [23].

We have assembled a combined list of missing proteins observed in mouse hepatocellular carcinoma from proteomic approaches. The database provides not only information on the nature of proteins present in mouse liver, but also potential protein diagnostic biomarkers to be examined in further investigations.

## CONCLUSIONS

The complete characterization of the human proteome is an ambitious task which is being carried out jointly by proteomics laboratories worldwide in the framework of the C-HPP project. In this study, we utilized a JAVA program by combining the HPA web database to reconstitute a missing protein search program. This software provides rapid analysis of suspected missing proteins for human chromosomes. We identified thousands of proteins by using the Mascot search base on the MS/MS data. A total of 116 proteins with high confidence level were in the GNMT−/− mouse model, of which 68 were newly identified missing proteins. We hope that this article will initiate the collaboration with the global C-HPP research team to quickly analyze mass spectrometry data for possible missing proteins.

## MATERIALS AND METHODS

### Mouse GNMT isolation and generation

A C57BL/6-strain mouse placental genomic DNA library constructed in lambda phage FIX II (Stratagene, La Jolla, CA) was used to isolate GNMT genomic clones. Human GNMT complementary DNA was used as a probe; hybridization procedures were performed according to standard protocols. A targeting vector was constructed and used to generate the GNMT knockout mouse model. The methods are detailed described previously [21]. All mice were kept in a 12-hour light-dark-cycle room with water and standard mouse pellet chow. The average age of mice sacrificed in this study was 15 months, and that of the tumor formation was around the age of 13 months. All the mice had been fasting for at least 8 hours before sacrifice. Each experiment was composed of eight to ten sets of animals. All animal experiments were carried out in accordance with the National Institutes of Health (NIH) Guide for the Care and Use of Laboratory Animals and approved by the Committee of Animal Use for Research at Kaohsiung Medical University.

### Serum alanine aminotransferase and aspartate aminotransferase tests

Serum samples without hemolysis were collected for determination of alanine aminotransferase (ALT) and aspartate aminotransferase (AST) activities. Liver transaminases were measured by Hitachi 7080 automatic biochemistry analyzer to be biomarkers of liver injury with some degree of intact liver function.

### Histopathological examination

Liver specimens were scored for fibrosis by examining Masson’s trichrome stained slides and all other parameters, such as degeneration/inflammation, were observed by examining H&E stained slides. The tissues were fixed in 10% formalin, processed, and embedded in the paraffin. The tissue slides were sectioned at 3–5 *μ*m in thickness, and stained with hematoxylin and eosin (H&E) or Masson’s Trichrome stain using standard procedure. The pathological reports were filed by the National Laboratory Animal Center.

### Sample digestion and preparation

The mouse livers were removed and homogenized, and then the proteins were extracted with RIPA buffer. Protein samples (100 μL) were transferred into 1.5 mL Eppendorf tubes and incubated at 37°C for 3 h after mixing with 25 μL of 1 M dithiothreitol (DTT, USB Corporation, 15397). The samples were reduced and alkylated in the dark, at room temperature, for 30 min after the addition of 25 μL of 1 M iodoacetamide (IAA, Amersham Biosciences, RPN6302V) in 25 mM ammonium bicarbonate. Approximately 10 μL of 0.1 μg/μL modified trypsin digestion buffer (Trypsin Gold, Mass Spectrometry Grade, V5280, Promega, WI, USA) in 25 mM ammonium bicarbonate was added to the protein samples, which were then incubated at 37°C for at least 12 h in a water bath. Two micro liters of formic acid were added to each sample before mass spectrometric analysis for protein identification.

### Proteome principal component analysis

The protein tryptic digest sample was prepared for Matrix-Assisted Laser Desorption/ Ionization Time of Flight Mass Spectrometry (MALDI-TOF/MS) analysis using a conventional dried droplet protocol. This protocol used α-cyano-4-hydroxycinnamic acid (CHCA, C8982, Sigma) as the matrix. The CHCA matrix was prepared as a saturated, aqueous solution containing 50% (v/v) acetonitrile (B15466, J.T. Baker) and 0.01% (v/v) trifluoroacetic acid (TFA, 302031, Aldrich). Each protein tryptic digest sample (1 μL) was dropped on the sample plate. Then 1:l of CHCA matrix was added to air-dry at room temperature.

MALDI-TOF/MS spectra were acquired using a MALDI-TOF mass spectrometer (Autoflex II, Bruker Daltonics, Bremen, Germany) running with the Flexcontrol Software Package (version 3). Generally, 500 laser shots were used, and the data of MALDI-TOF/MS were collected at different positions of each crystallized sample spot. For the proteome principal component analysis (PCA), all statistical analyses of MALDI-TOF/MS signals were analyzed by the ClinPro Tools with PCA software (version 3.0, Bruker Daltonics, Bremen, Germany). Statistical significance was evaluated at 95% of confidence level or better.

### Proteomic analysis

The protein tryptic digests were fractionated using a flow rate of 400 nL/min with a nano-UPLC system (nanoACQUITY UPLC, Waters, Milford, MA) coupled to an ion trap mass spectrometer (LTQ Orbitrap Discovery Hybrid FTMS, Thermo, San Jose, CA) equipped with an electrospray ionization source. For reverse phase nano-UPLC-ESI-MS/MS analyses, a sample (2 μL) of the desired peptide digest was loaded into the trapping column (Symmetry C18, 5 μm, 180 μm × 20 mm) by an autosampler. The reverse phase separation was performed using a linear acetonitrile gradient from 99% buffer A (100% D.I. water/0.1% formic acid) to 85% buffer B (100% acetonitrile/0.1% formic acid) in 100 min using the micropump at a flow rate of approximately 400 nL/min. The separation was performed on a C18 microcapillary column (BEH C18, 1.7 μm, 75 μm × 100 mm) using the nano separation system. As peptides were eluted from the micro-capillary column, they were electrosprayed into the ESI-MS/MS with the application of a distal 2.1 kV spraying voltage with heated capillary temperature of 200°C. Each scan cycle contained one full-scan mass spectrum (*m*/*z* range: 400–2000) and was followed by three data dependent tandem mass spectra. The collision energy of MS/MS analysis was set at 35%.

### Protein database search

Mascot software (Version 2.2.1, Matrix Science, London, UK) was used to search the Swiss-Prot protein sequence database. For proteolytic cleavages, only tryptic cleavage was allowed, and the number of maximal internal (missed) cleavage sites was set to 2. There was no modification allowed. Mass tolerances of the precursor peptide ion and fragment ion were set to 10 ppm and 0.5 Da, respectively. For missing protein identification, we downloaded all gene expression sequences from the human protein atlas web (HPA, www.proteinatlas.org). The search for the presence of any missing protein utilized JAVA programming based on the HPA. The peptide sequences performed by Mascot software were uploaded to our program for missing protein identification and the search algorithm was set to 100% match. When a protein was identified by two or more unique peptides, the protein was considered to be present in the sample.

## SUPPLEMENTARY MATERIALS TABLE




