# Supplementary material for: Utilizing glycine N-methyltransferasegene knockout mice as a model for identification of missing proteins in hepatocellular carcinoma
Source: Oncotarget. 2017 Dec 7;9(1):442–52. doi: 10.18632/oncotarget.23064 (PMC5787479; doi:10.18632/oncotarget.23064)
Supplement: Supplementary file 1 [file oncotarget-09-442-s001.pdf]

Utilizing glycine N-methyltransferasegene knockout mice as a model for identification of missing proteins in hepatocellular carcinoma

SUPPLEMENTARY MATERIALS

Supplementary Table 1: Unique peptides identified corresponding to missing proteins. See Supplementary_Table_1
