# Supplementary material for: Utilizing glycine N-methyltransferasegene knockout mice as a model for identification of missing proteins in hepatocellular carcinoma
Source: Oncotarget. 2017 Dec 7;9(1):442–52. doi: 10.18632/oncotarget.23064 (PMC5787479; doi:10.18632/oncotarget.23064)
Supplement: Supplementary file 2 [file oncotarget-09-442-s002.docx]

Table 1 – Unique peptides identified corresponding to missing proteins. List of 68 newly detected missing proteins in the mouse hepatocellular carcinoma (HCC) encoded on human chromosomes.

| **Group** | **Chromosome** | **Gene Name** | **UniProtKB Entry** | **UniProt protein name** | **Subcellular location** | **Molecular function** | **Biological process** | **Gene ID** | **Matched Sequence** |
| --- | --- | --- | --- | --- | --- | --- | --- | --- | --- |
| **I** | 1 | PPIAL4B | Q9Y536 | Peptidyl-prolyl cis-trans isomerase A-like 4A | Cytoplasm | [peptidyl-prolyl cis-trans isomerase activity](https://www.ebi.ac.uk/QuickGO/term/GO:0003755) | [protein folding](https://www.ebi.ac.uk/QuickGO/term/GO:0006457) | ENSG00000263353 | KTEWLDGK |
|  |  |  |  |  |  |  |  |  | RIIPGFMCQGGDFTR |
|  | 1 | PTCHD2-001 | Q9P2K9 | Protein dispatched homolog 3 | Endoplasmic reticulum membrane  Nucleus membrane  Cytoplasmic vesicle membrane Membrane |  | cholesterol homeostasis  cholesterol metabolic process  negative regulation of neuron differentiation  positive regulation of lipid metabolic process  positive regulation of neural precursor cell proliferation  regulation of lipid transport  smoothened signaling pathway | ENSG00000204624 | KLTACMSTVGLLQAASPSR |
|  |  |  |  |  |  |  |  |  | LVTIHEIE |
|  |  |  |  |  |  |  |  |  | LVTIHEIER |
|  |  |  |  |  |  |  |  |  | QHTRK |
|  | 1 | RP4-533D7.6 | F6TDL0 | Phosphatidylinositol 3-kinase regulatory subunit gamma | phosphatidylinositol 3-kinase complex | [1-phosphatidylinositol-3-kinase regulator activity](https://www.ebi.ac.uk/QuickGO/term/GO:0046935) | insulin receptor signaling pathway  phosphatidylinositol phosphorylation  positive regulation of glucose import in response to insulin stimulus  regulation of phosphatidylinositol 3-kinase activity | ENSG00000278139 | IMMNYD |
|  |  |  |  |  |  |  |  |  | IYHRDG |
|  |  |  |  |  |  |  |  |  | SKEYDRLYEEYT |
|  | 3 | KLHL24-001 | Q6TFL4 | Kelch-like protein 24 | Perikaryon Cell projection Cell junction |  | intermediate filament organization  protein autoubiquitination  protein ubiquitination  regulation of kainate selective glutamate receptor activity | ENSG00000114796 | QENCGMSVCNG |
|  |  |  |  |  |  |  |  |  | RYHILGNEMMSP |
|  |  |  |  |  |  |  |  |  | VQSYDPETNSWLL |
|  | 5 | PCDHA1 | Q9Y5I3 | Protocadherin alpha-1 | Isoform 1 : Cell membrane  Isoform 2 : Secreted | [calcium ion binding](https://www.ebi.ac.uk/QuickGO/term/GO:0005509) | cell adhesion  cell-cell signaling  homophilic cell adhesion via plasma membrane adhesion molecules  nervous system development | ENSG00000204970 | DSGANGQV |
|  |  |  |  |  |  |  |  |  | EETKK |
|  |  |  |  |  |  |  |  |  | EKGNSTTDNSD |
|  |  |  |  |  |  |  |  |  | GNSTTDNSDQ |
|  | 7 | AC068533.7 | H7C0S8 | Uncharacterized protein | cytoplasm | argininosuccinate lyase activity  DNA-directed 5'-3' RNA polymerase activity  nucleotide binding | arginine biosynthetic process via ornithine  transcription, DNA-templated | ENSG00000249319 | KAVFMAETK |
|  |  |  |  |  |  |  |  |  | KESGKN |
|  |  |  |  |  |  |  |  |  | KGMPFRQAHEASGK |
|  |  |  |  |  |  |  |  |  | KNPDSLELIR |
|  |  |  |  |  |  |  |  |  | RCAGLLMTLK |
|  |  |  |  |  |  |  |  |  | RDFVAEFLFWASLCMTHLSR |
|  |  |  |  |  |  |  |  |  | RQAHEASGKAVFMAETK |
|  | 12 | RP11-162P23.2 | F8VP50 | Uncharacterized protein |  | [aldehyde dehydrogenase (NAD) activity](https://www.ebi.ac.uk/QuickGO/term/GO:0004029) |  | ENSG00000257767 | KAARAAFQLGSPWR |
|  |  |  |  |  |  |  |  |  | KLGPALATGNVVVMK |
|  |  |  |  |  |  |  |  |  | KTIPIDGDFFSYTR |
|  |  |  |  |  |  |  |  |  | KVAEQTPLTALYVANLIK |
|  |  |  |  |  |  |  |  |  | RAAFQLGSPWR |
|  |  |  |  |  |  |  |  |  | RLADLIER |
|  | 13 | KLHL1 | Q9NR64 | Kelch-like protein 1 | Cytoplasm | [actin binding](https://www.ebi.ac.uk/QuickGO/term/GO:0003779) | actin cytoskeleton organization  adult walking behavior  cerebellar Purkinje cell layer development  dendrite development  protein ubiquitination | ENSG00000150361 | LQQGAPGQGTQQPA |
|  |  |  |  |  |  |  |  |  | NDLECQ |
|  |  |  |  |  |  |  |  |  | STVGTLYAVGGMDNN |
|  |  |  |  |  |  |  |  |  | TKPRKSTVGTLYAVGGMDNN |
|  |  |  |  |  |  |  |  |  | TLNTVECYNPKT |
|  | 17 | BAHCC1-201 | F8WBW8 | BAH and coiled-coil domain-containing protein 1 |  | [chromatin binding](https://www.ebi.ac.uk/QuickGO/term/GO:0003682) |  | ENSG00000266074 | KEALSFSKAKELSR |
|  |  |  |  |  |  |  |  |  | KEDSLLYAGSVR |
|  |  |  |  |  |  |  |  |  | KERQGLLGACR |
|  |  |  |  |  |  |  |  |  | KHLTSCLLNTK |
|  |  |  |  |  |  |  |  |  | KIQCTEPSPALLVSSSCRR |
|  |  |  |  |  |  |  |  |  | KLDHEGVTSPK |
|  |  |  |  |  |  |  |  |  | KNALYQSCHEDENDVQTISHK |
|  |  |  |  |  |  |  |  |  | KSRCLYPGNVVR |
|  |  |  |  |  |  |  |  |  | KWSGNPTQR |
|  |  |  |  |  |  |  |  |  | KWSGNPTQRR |
|  |  |  |  |  |  |  |  |  | RGSPLLSWSAVAQTK |
|  |  |  |  |  |  |  |  |  | RLTPRMQILQR |
|  |  |  |  |  |  |  |  |  | RQLWKWSGNPTQRR |
|  |  |  |  |  |  |  |  |  | RRGSPLLSWSAVAQTK |
|  |  |  |  |  |  |  |  |  | RSPARRGPGRPR |
|  | 18 | ASXL3-002 | Q9C0F0 | Putative Polycomb group protein ASXL3 | Nucleus | DNA binding  metal ion binding | regulation of transcription, DNA-templated  transcription, DNA-templated | ENSG00000141431 | QMGSDGIL |
|  |  |  |  |  |  |  |  |  | RNGVSMMVNKTVP |
|  |  |  |  |  |  |  |  |  | TLAHIKEQTKA |
|  |  |  |  |  |  |  |  |  | TWAEAARLALE |
|  |  |  |  |  |  |  |  |  | VSDEQSDSPSGSES |
|  | 19 | ZSWIM4-001 | Q9H7M6 | Zinc finger SWIM domain-containing protein 4 |  | [zinc ion binding](https://www.ebi.ac.uk/QuickGO/term/GO:0008270) |  | ENSG00000132003 | AKRVAE |
|  |  |  |  |  |  |  |  |  | EMLRMRDSNGA |
|  |  |  |  |  |  |  |  |  | LATLALAQLSIAFNQDSHPAVNDVLWACSLSHSLG |
|  |  |  |  |  |  |  |  |  | QCELASTMLTAA |
|  |  |  |  |  |  |  |  |  | QVELRLPISETLSQMNRDQLQ |
|  |  |  |  |  |  |  |  |  | VPFTRGLHLLQSGAVD |
| **II** | 1 | GLIS1 | Q8NBF1 | Zinc finger protein GLIS1 | Nucleus | metal ion binding  RNA polymerase II regulatory region sequence-specific DNA binding  transcriptional activator activity, RNA polymerase II transcription regulatory region sequence-specific binding  transcriptional repressor activity, RNA polymerase II transcription regulatory region sequence-specific binding | negative regulation of transcription from RNA polymerase II promoter  positive regulation of transcription from RNA polymerase II promoter  transcription from RNA polymerase II promoter | ENSG00000174332 | AFSNSSDRA |
|  |  |  |  |  |  |  |  |  | EQQVRK |
|  |  |  |  |  |  |  |  |  | HVKAHSA |
|  | 1 | ZBTB8B | Q8NAP8 | Zinc finger and BTB domain-containing protein 8B | Nucleus | DNA binding  metal ion binding | regulation of transcription, DNA-templated  transcription, DNA-templated | ENSG00000273274 | SHALSVH |
|  |  |  |  |  |  |  |  |  | TFTSHLSQGLR |
|  | 1 | ZSCAN20-004 | P17040 | Zinc finger and SCAN domain-containing protein 20 | Nucleus | DNA binding  metal ion binding  transcription factor activity, sequence-specific DNA binding | [transcription, DNA-templated](https://www.ebi.ac.uk/QuickGO/term/GO:0006351) | ENSG00000121903 | ESSS |
|  |  |  |  |  |  |  |  |  | RTHAGGKA |
|  | 2 | KLF7-201 | O75840 | Krueppel-like factor 7 | Nucleus | RNA polymerase II core promoter proximal region sequence-specific DNA binding  transcriptional activator activity, RNA polymerase II core promoter proximal region sequence-specific binding  transcription coactivator activity  transcription factor activity, sequence-specific DNA binding  zinc ion binding | axon guidance  dendrite morphogenesis  positive regulation of transcription from RNA polymerase II promoter  regulation of transcription from RNA polymerase II promoter | ENSG00000118263 | HLVKTSQTLSAVDGTVTL |
|  |  |  |  |  |  |  |  |  | TSQTLS |
|  | 3 | TIPARP-002 | Q7Z3E1 | TCDD-inducible poly [ADP-ribose] polymerase | Nucleus | enhancer binding  metal ion binding  NAD+ ADP-ribosyltransferase activity | androgen metabolic process  cellular response to organic cyclic compound  estrogen metabolic process  face morphogenesis  female gonad development  hemopoiesis  kidney development  multicellular organism metabolic process  negative regulation of gene expression  palate development  platelet-derived growth factor receptor signaling pathway  positive regulation of protein catabolic process  post-embryonic development  protein ADP-ribosylation  skeletal system morphogenesis  smooth muscle tissue development  vasculogenesis | ENSG00000163659 | HATMFGQGSYFA |
|  |  |  |  |  |  |  |  |  | IIYNLFH |
|  |  |  |  |  |  |  |  |  | RVQNQ |
|  | 4 | CCSER1-003 | Q9C0I3 | Serine-rich coiled-coil domain-containing protein 1 |  |  |  | ENSG00000184305 | LRSSSEGTAGSS |
|  |  |  |  |  |  |  |  |  | SSGST |
|  |  |  |  |  |  |  |  |  | SSSEGTAGSS |
|  | 4 | TMPRSS11F-001 | Q6ZWK6 | Transmembrane protease serine 11F | Membrane | [serine-type endopeptidase activity](https://www.ebi.ac.uk/QuickGO/term/GO:0004252) |  | ENSG00000198092 | KDVYDGLITPGMLCAGFMEG |
|  |  |  |  |  |  |  |  |  | MRNLLNS |
|  | 5 | CTC-432M15.3 | E9PCH4 | Uncharacterized protein | centrosome cytosol | [guanyl-nucleotide exchange factor activity](https://www.ebi.ac.uk/QuickGO/term/GO:0005085) | [small GTPase mediated signal transduction](https://www.ebi.ac.uk/QuickGO/term/GO:0007264) | ENSG00000273217 | LLNIACAAKAK |
|  |  |  |  |  |  |  |  |  | RYREP |
|  | 8 | SLC26A7-001 | Q8TE54 | Anion exchange transporter | Recycling endosome membrane | anion:anion antiporter activity  bicarbonate transmembrane transporter activity  chloride channel activity  oxalate transmembrane transporter activity  secondary active sulfate transmembrane transporter activity  sulfate transmembrane transporter activity | anion transport  bicarbonate transport  chloride transport  gastric acid secretion  ion transport  oxalate transport  regulation of intracellular pH  regulation of membrane potential  sulfate transport | ENSG00000147606 | SQSCP |
|  |  |  |  |  |  |  |  |  | TAGLYSTGA |
|  |  |  |  |  |  |  |  |  | TSQVKYL |
|  | 9 | CCDC183 | Q5T5S1 | Coiled-coil domain-containing protein 183 | axoneme |  | cilium movement  outer dynein arm assembly | ENSG00000213213 | KLIDKIHT |
|  |  |  |  |  |  |  |  |  | LNQQKKLID |
|  | 12 | EIF2S3L | Q2VIR3 | Putative eukaryotic translation initiation factor 2 subunit 3-like protein |  | GTPase activity  GTP binding  translation initiation factor activity |  | ENSG00000180574 | IDPTLC |
|  |  |  |  |  |  |  |  |  | KLSKNEVL |
|  | 16 | ZDHHC1-001 | Q8WTX9 | Probable palmitoyltransferase ZDHHC1 | Membrane | DNA binding  palmitoyltransferase activity  protein-cysteine S-palmitoyltransferase activity | [protein palmitoylation](https://www.ebi.ac.uk/QuickGO/term/GO:0018345) | ENSG00000159714 | CVCGFDHHC |
|  |  |  |  |  |  |  |  |  | MNICNKPSN |
| **III** | 5 | ADAMTS19 | Q8TE59 | A disintegrin and metalloproteinase with thrombospondin motifs 19 | Secreted | [metalloendopeptidase activity](https://www.ebi.ac.uk/QuickGO/term/GO:0004222)  [zinc ion binding](https://www.ebi.ac.uk/QuickGO/term/GO:0008270) |  | ENSG00000145808 | CSITCGKGMQS |
|  |  |  |  |  |  |  |  |  | GDFNHT |
|  |  |  |  |  |  |  |  |  | INVNTITSP |
|  |  |  |  |  |  |  |  |  | KCNEQPCQT |
|  |  |  |  |  |  |  |  |  | NLCQDMRWYQ |
|  |  |  |  |  |  |  |  |  | VMDGTSCGYQGLDICANG |
|  | 5 | RP11-449H3.3 | U3KPZ7 | Uncharacterized protein |  | [metal ion binding](https://www.ebi.ac.uk/QuickGO/term/GO:0046872)  [RNA binding](https://www.ebi.ac.uk/QuickGO/term/GO:0003723) | [mRNA processing](https://www.ebi.ac.uk/QuickGO/term/GO:0006397) | ENSG00000275740 | KIPQELNNIT |
|  |  |  |  |  |  |  |  |  | MLISKLEKN |
|  |  |  |  |  |  |  |  |  | NKNMKPEERANIM |
|  |  |  |  |  |  |  |  |  | RDQPG |
|  | 10 | WNT8B-001 | Q93098 | Protein Wnt-8b | Secreted | [frizzled binding](https://www.ebi.ac.uk/QuickGO/term/GO:0005109)  [receptor agonist activity](https://www.ebi.ac.uk/QuickGO/term/GO:0048018) | beta-catenin destruction complex disassembly  canonical Wnt signaling pathway  cell fate commitment  cellular response to retinoic acid  determination of dorsal identity  gastrulation  nervous system development  neuron differentiation  response to estradiol  response to retinoic acid  signal transduction  Wnt signaling pathway | ENSG00000075290 | AAMNLHNNEAG |
|  |  |  |  |  |  |  |  |  | ALQLSSHGGL |
|  |  |  |  |  |  |  |  |  | EKYHAALKVDLLQGAGNSAAG |
| **IV** | 1 | C1ORF127-001 | Q8N9H9 | Uncharacterized protein C1orf127 |  |  |  | ENSG00000175262 | ARRGQT |
|  |  |  |  |  |  |  |  |  | RQTPW |
|  | 1 | FAM110D | Q8TAY7 | Protein FAM110D |  |  |  | ENSG00000197245 | TPSAVE |
|  |  |  |  |  |  |  |  |  | VIQWLYGCQ |
|  | 1 | LINGO4 | Q6UY18 | Leucine-rich repeat and immunoglobulin-like domain-containing nogo receptor-interacting protein 4 | Membrane |  | [axonogenesis](https://www.ebi.ac.uk/QuickGO/term/GO:0007409) | ENSG00000213171 | DRGAYVCVVSNVAGNDSL |
|  |  |  |  |  |  |  |  |  | LSGNPLTCDC |
|  | 1 | PLD5-006 | Q8N7P1 | Inactive phospholipase D5. | Membrane | [catalytic activity](https://www.ebi.ac.uk/QuickGO/term/GO:0003824) |  | ENSG00000180287 | GAEVTYMNMTAYN |
|  |  |  |  |  |  |  |  |  | IREALVL |
|  |  |  |  |  |  |  |  |  | NCQNKC |
|  |  |  |  |  |  |  |  |  | QHEWLSASPHEGFEQM |
|  | 1 | SRGAP2B-001 | P0DMP2 | SLIT-ROBO Rho GTPase-activating protein 2B | Cytoplasm | [GTPase activator activity](https://www.ebi.ac.uk/QuickGO/term/GO:0005096)  [Rac GTPase binding](https://www.ebi.ac.uk/QuickGO/term/GO:0048365) | [negative regulation of cell migration](https://www.ebi.ac.uk/QuickGO/term/GO:0030336)  [nervous system development](https://www.ebi.ac.uk/QuickGO/term/GO:0007399) | ENSG00000196369 | FVQVSEDSGRLFK |
|  |  |  |  |  |  |  |  |  | QTPRSPDSTANV |
|  | 3 | ATP13A5-001 | Q4VNC0 | Probable cation-transporting ATPase 13A5 | Membrane | ATP binding  calcium-transporting ATPase activity  cation-transporting ATPase activity  metal ion binding | cellular calcium ion homeostasis  ion transmembrane transport | ENSG00000187527 | GMELI |
|  |  |  |  |  |  |  |  |  | LNYYVGMCGDGANDCGAL |
|  | 3 | GPR87-001 | Q9BY21 | G-protein coupled receptor 87 | Cell membrane | [G-protein coupled purinergic nucleotide receptor activity](https://www.ebi.ac.uk/QuickGO/term/GO:0045028) | [negative regulation of adenylate cyclase activity](https://www.ebi.ac.uk/QuickGO/term/GO:0007194) | ENSG00000138271 | SSRQFISQSSR |
|  |  |  |  |  |  |  |  |  | VNSCL |
|  | 5 | WNT8A | Q9H1J5 | Protein Wnt-8a | Secreted | [frizzled binding](https://www.ebi.ac.uk/QuickGO/term/GO:0005109)  [receptor agonist activity](https://www.ebi.ac.uk/QuickGO/term/GO:0048018) | beta-catenin destruction complex disassembly  canonical Wnt signaling pathway  canonical Wnt signaling pathway involved in cardiac muscle cell fate commitment  canonical Wnt signaling pathway involved in neural crest cell differentiation  neural crest cell fate commitment  neuron differentiation  palate development  regulation of transcription involved in anterior/posterior axis specification  response to retinoic acid  Wnt signaling pathway | ENSG00000061492 | ALMNLHNN |
|  |  |  |  |  |  |  |  |  | TCWLQLAEF |
|  | 5 | ZNF354B | Q96LW1 | Zinc finger protein 354B | Nucleus | chromatin binding  DNA binding  metal ion binding  transcription factor activity, sequence-specific DNA binding | negative regulation of transcription from RNA polymerase II promoter  regulation of transcription, DNA-templated  transcription, DNA-templated | ENSG00000178338 | ALSSHSTLIIHE |
|  |  |  |  |  |  |  |  |  | CSECGRAFSQSASLIQHE |
|  | 6 | FAM217A-001 | Q8IXS0 | Protein FAM217A |  |  |  | ENSG00000145975 | EIPVEQ |
|  |  |  |  |  |  |  |  |  | LQHMTIQ |
|  | 6 | PRDM13 | Q9H4Q3 | PR domain zinc finger protein 13 | Nucleus | chromatin binding  DNA binding  histone methyltransferase activity  metal ion binding | negative regulation of transcription from RNA polymerase II promoter  neurogenesis  transcription, DNA-templated | ENSG00000112238 | GGGGSGGSG |
|  |  |  |  |  |  |  |  |  | KYGLKIHM |
|  |  |  |  |  |  |  |  |  | MRTHT |
|  | 6 | TAAR5 | O14804 | Trace amine-associated receptor 5 | Cell membrane | G-protein coupled receptor activity  trimethylamine receptor activity | G-protein coupled receptor signaling pathway  sensory perception of chemical stimulus  signal transduction | ENSG00000135569 | IFVVATRQAQQI |
|  |  |  |  |  |  |  |  |  | YFNS |
|  | 7 | HOXA6 | P31267 | Homeobox protein Hox-A6 | Nucleus | [sequence-specific DNA binding](https://www.ebi.ac.uk/QuickGO/term/GO:0043565)  [transcription factor activity, sequence-specific DNA binding](https://www.ebi.ac.uk/QuickGO/term/GO:0003700) | [anterior/posterior pattern specification](https://www.ebi.ac.uk/QuickGO/term/GO:0009952)  [embryonic skeletal system morphogenesis](https://www.ebi.ac.uk/QuickGO/term/GO:0048704)  [transcription, DNA-templated](https://www.ebi.ac.uk/QuickGO/term/GO:0006351) | ENSG00000106006 | IEIANALCLTE |
|  |  |  |  |  |  |  |  |  | MNSCAGAVYGSHG |
|  |  |  |  |  |  |  |  |  | MNSCAGAVYGSHGRRG |
|  |  |  |  |  |  |  |  |  | TQPSGE |
|  | 7 | RASA4B-201 | C9J798 | Ras GTPase-activating protein 4B | Cytoplasm Cell membran | [GTPase activator activity](https://www.ebi.ac.uk/QuickGO/term/GO:0005096)  [metal ion binding](https://www.ebi.ac.uk/QuickGO/term/GO:0046872) | [intracellular signal transduction](https://www.ebi.ac.uk/QuickGO/term/GO:0035556)  [negative regulation of Ras protein signal transduction](https://www.ebi.ac.uk/QuickGO/term/GO:0046580) | ENSG00000170667 | CVNELNQWLSAL |
|  |  |  |  |  |  |  |  |  | SSPAGS |
|  |  |  |  |  |  |  |  |  | TATVW |
|  | 11 | ADAMTS15 | Q8TE58 | A disintegrin and metalloproteinase with thrombospondin motifs 15 | Secreted | [extracellular matrix binding](https://www.ebi.ac.uk/QuickGO/term/GO:0050840)  [heparin binding](https://www.ebi.ac.uk/QuickGO/term/GO:0008201)  [metalloendopeptidase activity](https://www.ebi.ac.uk/QuickGO/term/GO:0004222)  [zinc ion binding](https://www.ebi.ac.uk/QuickGO/term/GO:0008270) |  | ENSG00000166106 | FTRQD |
|  |  |  |  |  |  |  |  |  | YSGVSPRDKC |
|  | 12 | LHX5 | Q9H2C1 | LIM/homeobox protein Lhx5 | Nucleus | [metal ion binding](https://www.ebi.ac.uk/QuickGO/term/GO:0046872)  [sequence-specific DNA binding](https://www.ebi.ac.uk/QuickGO/term/GO:0043565) | cell proliferation in forebrain  cerebellar Purkinje cell differentiation  cerebellar Purkinje cell-granule cell precursor cell signaling involved in regulation of granule cell precursor cell proliferation  forebrain neuron differentiation  hippocampus development  positive regulation of transcription, DNA-templated  spinal cord association neuron differentiation  transcription, DNA-templated | ENSG00000089116 | SKVFHLNCFTCMVCNKQLSTGEELYVIDEN |
|  |  |  |  |  |  |  |  |  | TNLSEKCFS |
|  | 12 | MYRFL-003 | Q96LU7 | Myelin regulatory factor-like protein | Membrane | [DNA binding](https://www.ebi.ac.uk/QuickGO/term/GO:0003677)  [transcription factor activity, sequence-specific DNA binding](https://www.ebi.ac.uk/QuickGO/term/GO:0003700) |  | ENSG00000166268 | LHFSETTANNM |
|  |  |  |  |  |  |  |  |  | RSRSSE |
|  | 16 | MT4-001 | P47944 | Metallothionein-4 | cell | [metal ion binding](https://www.ebi.ac.uk/QuickGO/term/GO:0046872) | [cellular metal ion homeostasis](https://www.ebi.ac.uk/QuickGO/term/GO:0006875) | ENSG00000102891 | CARGCICKGGSD |
|  |  |  |  |  |  |  |  |  | GCICKGGSDKCSCC |
|  | 17 | OTOP2-201 | Q7RTS6 | Otopetrin-2 | Membrane |  |  | ENSG00000183034 | HTLSA |
|  |  |  |  |  |  |  |  |  | RAMDHHKNPT |
|  | 19 | BEST2-002 | Q8NFU1 | Bestrophin-2 | Cell membrane | [chloride channel activity](https://www.ebi.ac.uk/QuickGO/term/GO:0005254) | [membrane depolarization](https://www.ebi.ac.uk/QuickGO/term/GO:0051899)  [sensory perception of smell](https://www.ebi.ac.uk/QuickGO/term/GO:0007608) | ENSG00000039987 | MTVTYTARVANA |
|  |  |  |  |  |  |  |  |  | QFLDPAQGY |
|  | 19 | NTN5-001 | Q8WTR8 | Netrin-5 | Secreted |  |  | ENSG00000142233 | LGVTGLTCNRCGPGYQQS |
|  |  |  |  |  |  |  |  |  | LGVTGLTCNRCGPGYQQSRSP |
|  | 19 | ZFP14-001 | Q9HCL3 | Zinc finger protein 14 homolog | Nucleus | [DNA binding](https://www.ebi.ac.uk/QuickGO/term/GO:0003677)  [metal ion binding](https://www.ebi.ac.uk/QuickGO/term/GO:0046872)  [transcription factor activity, sequence-specific DNA binding](https://www.ebi.ac.uk/QuickGO/term/GO:0003700) | [regulation of transcription, DNA-templated](https://www.ebi.ac.uk/QuickGO/term/GO:0006355)  [transcription, DNA-templated](https://www.ebi.ac.uk/QuickGO/term/GO:0006351) | ENSG00000142065 | AFRQHSHLTQHQ |
|  |  |  |  |  |  |  |  |  | GMVVR |
|  | 20 | ADRA1D-001 | P25100 | Alpha-1D adrenergic receptor | Cell membrane | [alpha1-adrenergic receptor activity](https://www.ebi.ac.uk/QuickGO/term/GO:0004937) | adenylate cyclase-activating adrenergic receptor signaling pathway  adenylate cyclase-modulating G-protein coupled receptor signaling pathway  cell-cell signaling  cell proliferation  DNA metabolic process  G-protein coupled receptor signaling pathway  multicellular organism development  norepinephrine-epinephrine vasoconstriction involved in regulation of systemic arterial blood pressure  phospholipase C-activating G-protein coupled receptor signaling pathway  positive regulation of cell proliferation  positive regulation of cytosolic calcium ion concentration  positive regulation of smooth muscle contraction  positive regulation of vasoconstriction | ENSG00000171873 | HSLKYPAIMTE |
|  |  |  |  |  |  |  |  |  | VSSLSH |
|  | 20 | NNAT-001 | Q16517 | Neuronatin | Cytoplasm |  | brain development  neuron differentiation  positive regulation of insulin secretion  protein lipoylation  transport | ENSG00000053438 | QVLGER |
|  |  |  |  |  |  |  |  |  | YSLQKL |
|  | 23X | NEXMIF | Q5QGS0 | Neurite extension and migration factor | Nucleus Cytoplasm |  | [error-prone translesion synthesis](https://www.ebi.ac.uk/QuickGO/term/GO:0042276)  [nervous system development](https://www.ebi.ac.uk/QuickGO/term/GO:0007399) | ENSG00000050030 | APFAIMEPAGMSALNGDCLMQPSRTCLGCFMES |
|  |  |  |  |  |  |  |  |  | ESTDLLDISNFTPDKF |
|  |  |  |  |  |  |  |  |  | YMARDTNSGSSSSQQNYGLRA |
|  | 23X | PTCHD1-201 | Q96NR3 | Patched domain-containing protein 1 | Cell membrane |  | [cognition](https://www.ebi.ac.uk/QuickGO/term/GO:0050890)  [smoothened signaling pathway](https://www.ebi.ac.uk/QuickGO/term/GO:0007224)  [social behavior](https://www.ebi.ac.uk/QuickGO/term/GO:0035176)  [thalamus development](https://www.ebi.ac.uk/QuickGO/term/GO:0021794) | ENSG00000165186 | ATNRTNFAITYPITHL |
|  |  |  |  |  |  |  |  |  | TNFAITYPITHLKDG |
| **V** | 1 | ETV3L | Q6ZN32 | ETS translocation variant 3-like protein | Nucleus | RNA polymerase II transcription factor activity, sequence-specific DNA binding  sequence-specific DNA binding  transcriptional repressor activity, RNA polymerase II transcription regulatory region sequence-specific binding | cell differentiation  regulation of transcription from RNA polymerase II promoter  transcription, DNA-templated | ENSG00000253831 | AESSPGS |
|  |  |  |  |  |  |  |  |  | HVIAWQQGEYGEFVI |
|  | 1 | LRRC53 | A6NM62 | Leucine-rich repeat-containing protein 53 | Membrane |  |  | ENSG00000162621 | FMKTN |
|  |  |  |  |  |  |  |  |  | KTDSS |
|  | 1 | LRRN2-003 | O75325 | Leucine-rich repeat neuronal protein 2 | Membrane | [receptor activity](https://www.ebi.ac.uk/QuickGO/term/GO:0004872) | axonogenesis  cell adhesion  signal transduction | ENSG00000170382 | LALAVL |
|  |  |  |  |  |  |  |  |  | NLLRLHLNSN |
|  | 2 | RGPD3-201 | A6NKT7 | RanBP2-like and GRIP domain-containing protein 3 | Intracellular |  | [intracellular transport](https://www.ebi.ac.uk/QuickGO/term/GO:0046907) | ENSG00000153165 | ERGIGDIKILQNYDN |
|  |  |  |  |  |  |  |  |  | GSLRN |
|  |  |  |  |  |  |  |  |  | LSQSGHMLLNLS |
|  |  |  |  |  |  |  |  |  | WNSCV |
|  | 2 | SP5 | Q6BEB4 | Transcription factor Sp5 | Nucleus | metal ion binding  RNA polymerase II regulatory region sequence-specific DNA binding  RNA polymerase II transcription factor activity, sequence-specific DNA binding | bone morphogenesis  cellular response to organic cyclic compound  negative regulation of transcription from RNA polymerase II promoter  post-anal tail morphogenesis  regulation of transcription from RNA polymerase II promoter  transcription, DNA-templated | ENSG00000204335 | NDSLQAFLQDRTPSASPDLG |
|  |  |  |  |  |  |  |  |  | TPSASPDLGKHSPLALLAATCS |
|  | 3 | KCNH8-001 | Q96L42 | Potassium voltage-gated channel subfamily H member 8 | Membrane | [phosphorelay sensor kinase activity](https://www.ebi.ac.uk/QuickGO/term/GO:0000155)  [voltage-gated potassium channel activity](https://www.ebi.ac.uk/QuickGO/term/GO:0005249) | [regulation of membrane potential](https://www.ebi.ac.uk/QuickGO/term/GO:0042391) | ENSG00000183960 | EGHESDVISRLSN |
|  |  |  |  |  |  |  |  |  | ETNEQ |
|  |  |  |  |  |  |  |  |  | MLEYFQTTWSVNNGIDSNELL |
|  | 4 | NKX1-1-001 | Q15270 | NK1 transcription factor-related protein 1 | Nucleus | [sequence-specific DNA binding](https://www.ebi.ac.uk/QuickGO/term/GO:0043565) | lipid metabolic process  nervous system process  regulation of generation of precursor metabolites and energy  regulation of glucose metabolic process  regulation of transcription, DNA-templated | ENSG00000235608 | DSGDE |
|  |  |  |  |  |  |  |  |  | YLSVCE |
|  | 8 | NKX6-3-001 | A6NJ46 | Homeobox protein Nkx-6.3 | Nucleus | [sequence-specific DNA binding](https://www.ebi.ac.uk/QuickGO/term/GO:0043565)  [transcriptional activator activity, RNA polymerase II transcription regulatory region sequence-specific binding](https://www.ebi.ac.uk/QuickGO/term/GO:0001228) | [positive regulation of transcription from RNA polymerase II promoter](https://www.ebi.ac.uk/QuickGO/term/GO:0045944) | ENSG00000165066 | AGNEYPTRT |
|  |  |  |  |  |  |  |  |  | SALEPSSSTP |
|  | 8 | SGCZ-001 | Q08AT0 | Submitted name: SGCZ protein | Plasma Membrane |  |  | ENSG00000185053 | DSPLVLQSD |
|  |  |  |  |  |  |  |  |  | KDSPLVLQSD |
|  |  |  |  |  |  |  |  |  | SSSPSSS |
|  | 9 | SPATA31A1 | Q5TZJ5 | Spermatogenesis-associated protein 31A1 | Membrane Curated |  | [cell differentiation](https://www.ebi.ac.uk/QuickGO/term/GO:0030154)  [spermatogenesis](https://www.ebi.ac.uk/QuickGO/term/GO:0007283) | ENSG00000204849 | SAEAQ |
|  |  |  |  |  |  |  |  |  | VRRSWL |
|  | 11 | RASSF10 | A6NK89 | Ras association domain-containing protein 10 | Cytoplasm |  | [signal transduction](https://www.ebi.ac.uk/QuickGO/term/GO:0007165) | ENSG00000189431 | ILPNKT |
|  |  |  |  |  |  |  |  |  | RTTCSDVV |
|  | 12 | MGAT4C-006 | Q9UBM8 | Alpha-1,3-mannosyl-glycoprotein 4-beta-N-acetylglucosaminyltransferase C | Golgi apparatus membrane | [alpha-1,3-mannosylglycoprotein 4-beta-N-acetylglucosaminyltransferase activity](https://www.ebi.ac.uk/QuickGO/term/GO:0008454)  [metal ion binding](https://www.ebi.ac.uk/QuickGO/term/GO:0046872) | [carbohydrate metabolic process](https://www.ebi.ac.uk/QuickGO/term/GO:0005975)  [protein glycosylation](https://www.ebi.ac.uk/QuickGO/term/GO:0006486) | ENSG00000182050 | ETSTHQLNSE |
|  |  |  |  |  |  |  |  |  | ETSTHQLNSERYVHTF |
|  |  |  |  |  |  |  |  |  | NFLTAIK |
|  | 12 | RP11-302B13.5 | F5H423 | Uncharacterized protein | Intracellular | [GTP binding](https://www.ebi.ac.uk/QuickGO/term/GO:0005525) | [small GTPase mediated signal transduction](https://www.ebi.ac.uk/QuickGO/term/GO:0007264) | ENSG00000272822 | EMRILMVGLDAAGKTTILY |
|  |  |  |  |  |  |  |  |  | MGNIFGNLLK |
|  |  |  |  |  |  |  |  |  | MLAEDELRDAVLLVFAN |
|  |  |  |  |  |  |  |  |  | RILMVGLDAAGK |
|  |  |  |  |  |  |  |  |  | RMLAEDELRDAVLLVFANK |
|  | 12 | ZNF664-009 | Q8N3J9 | Zinc finger protein 664 | Nucleus | [DNA binding](https://www.ebi.ac.uk/QuickGO/term/GO:0003677)  [metal ion binding](https://www.ebi.ac.uk/QuickGO/term/GO:0046872) | [regulation of transcription, DNA-templated](https://www.ebi.ac.uk/QuickGO/term/GO:0006355)  [transcription, DNA-templated](https://www.ebi.ac.uk/QuickGO/term/GO:0006351) | ENSG00000179195 | AFRHTSSLCMHQ |
|  |  |  |  |  |  |  |  |  | DFSTTTKLN |
|  | 14 | C14ORF28-001 | Q4W4Y0 | Uncharacterized protein C14orf28 |  |  |  | ENSG00000179476 | ILLNK |
|  |  |  |  |  |  |  |  |  | TLFEEIKASIKNNYNQD |
|  | 20 | SCRT2-001 | Q9NQ03 | Transcriptional repressor scratch 2 | Nucleus | [DNA binding](https://www.ebi.ac.uk/QuickGO/term/GO:0003677)  [metal ion binding](https://www.ebi.ac.uk/QuickGO/term/GO:0046872)  [transcriptional repressor activity, RNA polymerase II core promoter proximal region sequence-specific binding](https://www.ebi.ac.uk/QuickGO/term/GO:0001078) | negative regulation of extrinsic apoptotic signaling pathway via death domain receptors  negative regulation of transcription from RNA polymerase II promoter  regulation of neuron migration  transcription, DNA-templated | ENSG00000215397 | CRQCDKSFAL |
|  |  |  |  |  |  |  |  |  | QTHRSLDSQLAR |

Protein functions described in UniProt databases (http://www.uniprot.org/uniprot/). Some proteins listed in Table 1 still have no clear known subcellular location and functions to date.
